# A Membrane Permeable Prodrug of S223 for Selective Epac2 Activation in Living Cells

**DOI:** 10.3390/cells8121589

**Published:** 2019-12-06

**Authors:** Yunjian Xu, Frank Schwede, Hans Wienk, Anders Tengholm, Holger Rehmann

**Affiliations:** 1 Department of Medical Cell Biology, Uppsala University, Biomedical Centre, Box 571, SE-75123 Uppsala, Sweden; yunjian.xu@mcb.uu.se (Y.X.); anders.tengholm@mcb.uu.se (A.T.); 2 BIOLOG Life Science Institute, Flughafendamm 9a, 28199 Bremen, Germany; fs@biolog.de; 3 NMR Spectroscopy, Bijvoet Center for Biomolecular Research, Utrecht University, Padualaan 8, 3584 CH Utrecht, The Netherlands; h.wienk@nki.nl; 4 Department of Molecular Cancer Research, Center for Molecular Medicine, Oncode Institute, University Medical Center Utrecht, 3584 CX Utrecht, The Netherlands

**Keywords:** cAMP, Epac, PKA, prodrug, nucleotide analogue, acetoxymethyl ester, insulin secretion, islets, pancreatic β-cell, Rap

## Abstract

Signalling by cyclic adenosine monophosphate (cAMP) occurs via various effector proteins, notably protein kinase A and the guanine nucleotide exchange factors Epac1 and Epac2. These proteins are activated by cAMP binding to conserved cyclic nucleotide binding domains. The specific roles of the effector proteins in various processes in different types of cells are still not well defined, but investigations have been facilitated by the development of cyclic nucleotide analogues with distinct selectivity profiles towards a single effector protein. A remaining challenge in the development of such analogues is the poor membrane permeability of nucleotides, which limits their applicability in intact living cells. Here, we report the synthesis and characterisation of S223-AM, a cAMP analogue designed as an acetoxymethyl ester prodrug to overcome limitations of permeability. Using total internal reflection imaging with various fluorescent reporters, we show that S223-AM selectively activates Epac2, but not Epac1 or protein kinase A, in intact insulin-secreting β-cells, and that this effect was associated with pronounced activation of the small G-protein Rap. A comparison of the effects of different cAMP analogues in pancreatic islet cells deficient in Epac1 and Epac2 demonstrates that cAMP-dependent Rap activity at the β-cell plasma membrane is exclusively dependent on Epac2. With its excellent selectivity and permeability properties, S223-AM should get broad utility in investigations of cAMP effector involvement in many different types of cells.

## 1. Introduction

The second messenger cyclic adenosine monophosphate (cAMP) acts via binding to protein kinase A (PKA), cyclic nucleotide regulated ion channels, and the guanine nucleotide exchange factors (GEFs) Epac1 and Epac2, thereby controlling a wide variety of physiological processes ranging from cell proliferation and differentiation to electrical activity and hormone secretion [1,2]. Epac1 and Epac2 are GEFs for the small G-proteins of the Rap family [3,4], and might also act by mediating protein–protein interactions [5]. While PKA and Epac1 are expressed ubiquitously, the expression of Epac2 is rather restricted to the pancreas and the brain [3]. In pancreatic β-cells, cAMP is an important amplifier of insulin exocytosis, and the messenger mediates the insulinotropic action of glucagon and the incretin hormone glucagon-like peptide-1 [6]. cAMP-dependent effects on insulin secretion are mediated by both PKA and Epac [7,8,9,10,11]. Epac1 seems to play a role primarily for normal development of the pancreatic islets [12] but is expressed at much lower levels than Epac2 in β-cells [13,14]. A function of Epac2 in insulin secretion was first proposed based on its interaction with ATP-sensitive potassium channels, which link glucose metabolism to electrical activity in β-cells, and with Rim2, a protein involved in secretory granule docking and priming [15]. It was subsequently suggested that Epac2 enhances secretion by increasing the cytoplasmic Ca^2+^ concentration [16,17], potentially via PLCε [18], by increasing the density of insulin granules at the plasma membrane [19], and by priming them for exocytosis [20]. cAMP elevations induce translocation of Epac2 to the β-cell plasma membrane [21] and in particular to secretory granule docking sites [22], where the protein stabilises the fusion pore during exocytosis [23]. Epac2-selective agonists would be valuable tools in unravelling the detailed mechanisms of the action of Epac2 in β-cells and other types of cells.

The activation mechanism of the Epac proteins by cAMP is well understood at the molecular level [24,25]. Epac exists in dynamic equilibrium between an inactive and an active conformation, which is shifted by cAMP binding to the active site [26,27]. However, the cAMP-induced shift in equilibrium is not complete, and a fraction of Epac remains in the inactive conformation also when bound to cAMP. When describing the agonistic potency of cAMP and synthetic nucleotide analogues, there are consequently two biophysical parameters to consider, which can be determined by the use of recombinant Epac in vitro [27]. The first of these parameters is the affinity with which the analogue binds. The second parameter quantifies the degree to which the equilibrium is shifted to the active conformation, measured as the maximal activity that can be achieved under saturation. For example, the cAMP analogue D007 has a 10-fold higher affinity for Epac1 and induces three times higher maximal activity compared to native cAMP [26]. D007 is therefore considered as a “superactivator” of Epac1. D007 was the first cAMP analogue described as being capable of discriminating Epac from PKA [28]. However, it is important to realise that D007 has the potential to activate PKA, although its affinity is greatly reduced compared to unmodified cAMP [29]. 

Even though D007 had been biophysically characterised only with Epac1, the cAMP analogue was used for many years without differentiation between Epac1 and Epac2 in experimental design or data interpretation. Only recently, it was found that D007 is in fact a relatively poor activator of Epac2, with an affinity comparable to that of cAMP and a maximal activation capacity lower than that of the natural agonist [30]. Indeed, D007 only very weakly activated Rap in Epac2-expressing cells deficient in Epac1 [30]. Using a structure-guided approach, another analogue, S220, was developed as a potent activator of Epac2 [30]. Compared to cAMP, S220 has a 10 times higher affinity for Epac2 and induces seven times higher maximal activity [30]. In contrast, its maximal activity towards Epac1 is only one third of that of cAMP. Unlike D007, S220 activates PKA in vitro with only slightly reduced affinity compared to cAMP. This effect may be less pronounced in cells and when evaluated in intact U2OS cells, S220 activated Epac2 with little effect on Epac1 or PKA [30]. The S220 derivate S223 shows a drastically improved discrimination between Epac2 and PKA [30]. While its activity towards PKA is negligible, S223 has a similar affinity for Epac2 as cAMP, but induces a three times higher maximal activity. Thus, S223 is a weaker Epac2 agonist than S220. Its activation of Epac1 is comparable with that of S220. In contrast to the promising properties of S223 as an Epac2-selective agonist in vitro, the analogue disappointingly failed to activate Rap when tested with intact cells [30].

The applicability of cAMP analogues in living cells is strongly limited by their low membrane permeability, which is mainly a consequence of the negatively charged phosphate. This limitation can be overcome by converting the cAMP analogue into a prodrug, in which a masking group is attached to the phosphate. Such a prodrug is uncharged, but upon entry into the cytoplasm, the masking group is cleaved off whereby the charged mother compound is released. The conversion of phosphate groups into acetoxymethyl (AM) esters is a common prodrug strategy [31], which was successfully used to increase the accessibility of D007 to intact cells [32]. Therefore, we tested if generation of an analogous membrane-permeable prodrug of S223 would enable its use as a selective Epac2 activator in intact, living cells. Here, we describe the synthesis and characterisation of such a membrane-permeable AM ester of S223 in vitro as well as in cell lines and intact pancreatic islets.

## 2. Materials and Methods

### 2.1. NMR

S223-AM was dissolved in dried deuterated DMSO. Spectra were recorded at 293 K on a 750 MHz Bruker Avance NMR machine equipped with 5 mm QXI probe. Reported chemical shifts are calibrated directly (^1^H) or indirectly (^31^P, ^13^C) with respect to DSS. Standard processing and assignments were performed with Topspin and Sparky, combining ^1^D ^1^H, ^13^C, DEPT and ^31^P, and ^2^D ROESY, COSY, TOCSY (mixing times of 20, 100 and 200 ms), [^1^H;^31^P]-HSQC and [^1^H;^13^C]-HSQC experiments.

### 2.2. Hydrolysis of S223-AM

S223-AM was incubated at a concentration of 50 µM in the different matrices in a total volume of 200 µL in a thermomixer set at 300 rpm and 37 °C. Samples in PBS were supplemented with 25% CH_3_CN to avoid precipitation of S223-AM. All other samples were supplemented with 5% CH_3_CN. Samples were removed and immediately injected into the HPLC systems without any further operations to remove cellular or buffer contents to avoid degradation of cyclic nucleotides during sample preparation. The two separate analytical isocratic HPLC-systems were operated in parallel, one for S223-AM detection and one for S223 and OXO detection. The systems consisted of a L-6200 pump, a L-4250 variable wavelength UV/Vis detector (set at 283 nm), and a D-7500 GPC integrator (all Merck-Hitachi, Darmstadt, Germany). The stationary phases were Kromasil 100-10 C8, 10 µm (Ziemer Chromatographie, Langerwehe, Germany) in 250 × 4.6 mm stainless steel columns with Gemini C18, 10 × 10 mm Security guardTM columns (Phenomenex, Aschaffenburg, Germany). All chromatographic operations were performed at ambient temperature. For S223-AM quantification, 52% CH_3_CN, 1 mM triethylammonium formate (TEAF), pH 6 was used as mobile phase with a flow rate of 1 mL/min and for S223 and OXO quantification, 28% CH_3_CN, 20 mM TEAF, 2 mM NaH_2_PO_4_, pH 6.3 with a flow rate of 1.5 mL/min.

### 2.3. U2OS Cell Model System

The generation and characterisation of U2OS cells stably expressing Epac1 and Epac2 and the protocols to monitor Epac and PKA activities have been described previously [30]. In brief, after 20 min of stimulation, cells were lysed and Rap•GTP levels determined by precipitating GTP-bound Rap and Western blot analysis with a α-Rap antibody (Santa Cruz Biotechnology, USA). To determine PKA activity, total cell lysates were subjected to Western blotting with a monoclonal α-VASP antibody (BD Transduction Laboratories, USA).

### 2.4. Insulin-Secreting MIN6 β-cells

The murine insulin-secreting β-cell line MIN6 [33] was cultured as previously described [22]. Transfection with plasmid DNA for fluorescent cell signaling biosensors or Epac fluorescent protein fusions was carried out while seeding the cells onto 25-mm coverslips coated with poly-L-lysine. A suspension of 0.2 million cells in Optimen I medium (Invitrogen) containing Lipofectamine 2000 (Invitrogen) and plasmid DNA was placed onto the centre of each coverslip. The cells were allowed to attach during 4 h incubation before the transfection was interrupted by addition of 3 mL complete cell culture medium. Cells were maintained in this medium for 24 h to allow expression of the fluorescent protein constructs before imaging. 

### 2.5. Pancreatic Islets

Pancreatic islets were isolated from wildtype and globally Epac-deficient C57Bl/6J mice [34] bred in-house. All the procedures for animal handling and islet isolation were approved by the Uppsala animal ethics committee. The islets were cultured for 1–2 days in RPMI 1640 medium containing 5.5 mM glucose, 10% calf serum, 100 U/mL penicillin and 100 µg/mL streptomycin at 37 °C in a humidified air atmosphere with 5% CO_2_. The islets were infected for 1–2 h with Rap biosensor-expressing adenovirus [21] at a concentration of 10^5^ fluorescence forming units per islet, followed by washing and further culture for 16–20 h before use. 

### 2.6. Total Internal Reflection Fluorescence Imaging of Epac Translocation and Plasma Membrane Activities of Rap and PKA

The cells or islets expressing fluorescent protein constructs were preincubated for 30 min in experimental buffer containing (in mM) NaCl 138, KCl 4.8, CaCl_2_ 1.3, MgCl_2_ 1.2, glucose 3, HEPES 25 (pH 7.40) and 1 mg/mL albumin. The islets were subsequently transferred to a poly-L-lysine-coated coverslip and allowed to attach during 5 min. The coverslips with cells or islets were used as exchangeable bottoms of an open chamber continuously superfused with medium at 37 °C. The chamber was mounted on the thermostated stage of a total internal reflection fluorescence (TIRF) microscope. Whereas experiments on MIN6 cells were performed on a prism-based system, imaging of intact islets were performed on an objective-based setup, essentially as previously described [21]. The prism TIRF system was built around an E600FN upright microscope (Nikon, Tokyo, Japan) with a 16×, 0.8-NA water immersion objective. A HeCd laser (Kimmon Koha, Tokyo, Japan) provided 442 nm light for excitation of CFP and diode-pumped solid-state lasers provided 491 and 515 nm light for excitation of GFP and YFP (Cobolt, Stockholm, Sweden). Emission light was detected with a back-illuminated EMCCD camera (DU897, Andor Technology, Belfast, Northern Ireland, UK) at 483 nm/32 nm half-bandwidth for CFP, 530/35 nm for GFP and 542/27 nm for YFP. The objective-based setup utilized an inverted Ti microscope equipped with a 60×, 1.45-NA objective and a TIRF illuminator (Nikon). A diode-pumped solid-state laser (Cobolt) provided excitation light at 491 nm. Emission was detected at 530/50 nm using a digital camera (Orca AG, Hamamatsu Photonics, Hamamatsu City, Japan). Time-lapse recordings were performed with image or image-pair acquisition every 5 s using MetaFluor software (Molecular Devices, Downington, PA, USA). 

The activity-dependent translocation of Epac proteins from the cytoplasm to the plasma membrane was recorded in MIN6 cells using GFP-Epac2 and YFP-Epac1 constructs as previously described [22]. The plasma membrane activity of Rap was recorded using a GFP-tagged RBD-domain from RalGDS (GFP-RalGDS^RBD^) as described in [21]. Changes in Epac or Rap biosensor binding to the plasma membrane were recorded as changes in TIRF intensity and expressed as the fluorescence intensity (F) normalized to the prestimulatory intensity (F_0_). In MIN6 cells, the Rap biosensor signal was divided by that of a co-transfected red fluorescent protein membrane marker (td2-CAAX, [21]) and the resulting ratio signal (R) normalized to the prestimulatory ratio (R_0_).

PKA activity was recorded using a plasma-membrane anchored version of AKAR3 (kind gift from Prof. Jin Zhang, University of California, San Diego, USA). A PKA phosphorylation-induced conformational change of the sensor is detected as increased fluorescence resonance energy transfer (FRET) between CFP and YFP. AKAR3 was excited at 442 nm with donor emission detected at 483/32 nm and sensitized acceptor emission at 542/27 nm. FRET was recorded as the 542/483 nm fluorescence ratio (FRET ratio) normalized to the prestimulatory ratio. 

Quantifications of the magnitudes of the fluorescence or ratio changes were made by measuring the area under the curve during the stimulation period, subtracting the baseline defined as the level immediately preceding each stimulation, and finally normalizing for the elapsed time to obtain the time-averaged response.

If not otherwise stated, the following concentrations of the drugs were used in the live-cell imaging experiments: S223-AM 5 µM, S220 100 µM, D007-AM 5 µM, IBMX 100 µM and forskolin 10 µM. The AM ester versions of the cyclic nucleotide analogues were used at lower concentrations than the non-esterified analogue, due to their expected higher membrane permeability [32].

## 3. Results

### 3.1. Synthesis of S223-AM

**Figure 1:**
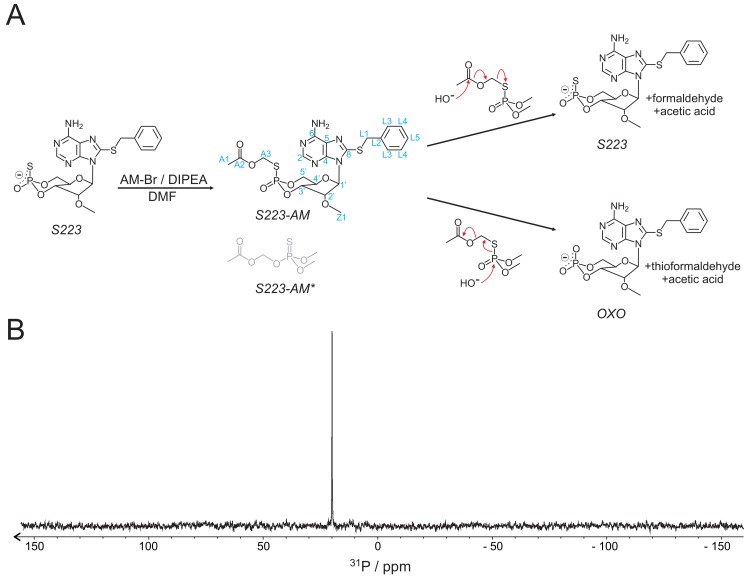
Synthesis of S223-AM. (**A**) Scheme of synthesis and hydrolysis of S223-AM. The reaction is performed with acetoxymethyl bromide (AM-Br) as a donor of the acetoxymethyl group in the presence of *N*,*N*-diisopropylethylamine (DIPEA) in N,N-dimethylformamide (DMF). It results only in the S-linked (S223-AM) but not the O-linked (S223-AM*) version. The postulated mechanisms of hydrolysis resulting in either S223 or OXO are shown. Blue labels, atom identities. (**B**) ^31^P-NMR spectrum of S223-AM recorded in DMSO.

The AM ester of S223 was synthesised as shown in Figure 1A, following protocols described in [32]. The molecular identity was confirmed by ^1^H, ^13^C, and ^31^P NMR spectroscopy (Figure 1B and Table 1). As sulphur is a better nucleophile than oxygen, it is expected that coupling of the AM-group via the sulphur was favourable and thus dominates the product. In fact, only one species was obtained as demonstrated by a single signal in the ^31^P-NMR spectrum and a single set of NMR signals for the protons and carbons of the AM-group. Thus, in the obtained product, the AM group is exclusively coupled to the sulphur. This finding was further confirmed by studies on the enzymatic hydrolysis of the ester (see below). 

### 3.2. Stability and Hydrolysis Mechanism of S223-AM

The intended use of S223-AM for applications in cells, organs or even in vivo, requires that the ester be sufficiently stable in aqueous environment but can be readily hydrolysed intracellularly by a mechanism that releases S223. The hydrolysis of S223-AM by an attacking nucleophile, typically a water molecule or hydroxide anion, can occur by two different mechanisms (Figure 1A). The nucleophile either attacks on the carbon of the acetoxy-group or on the phosphate. An attack on the acetoxy-group results in the release of S223 and formaldehyde and acetic acid as by-products. Attack on the phosphate, however, results in cleavage of the phosphate–sulphur bond and thereby, the generation of OXO and thioformaldehyde and acetic acid as by-products. Hydrolysis of S223-AM by attack on the phosphate is unwanted, as this reaction eliminates the sulphur from the released nucleotide. This sulphur in the axial position contributes to strong Epac2 activation [30].

**Figure 2:**
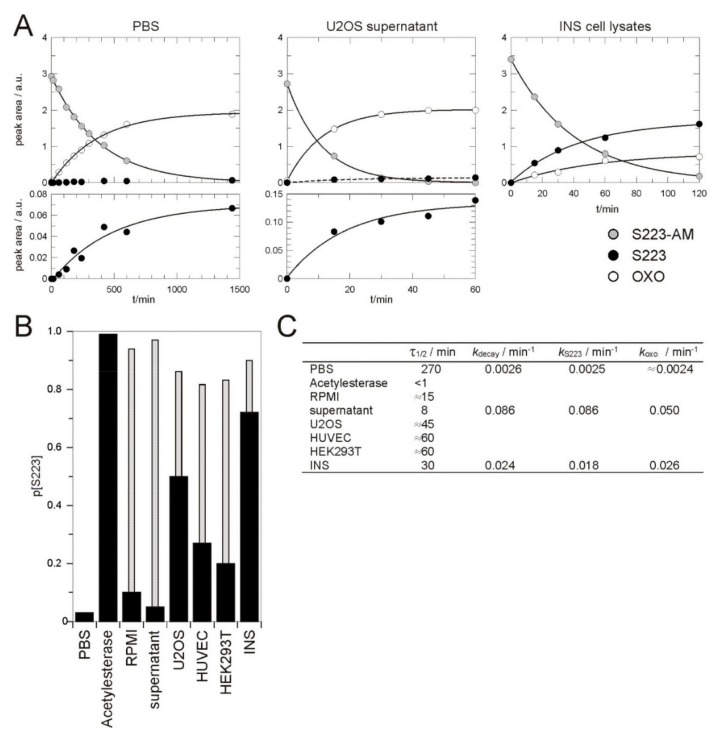
Hydrolysis of S223-AM. (**A**) S223-AM was exposed to PBS (left), U2OS cell culture supernatant (middle), and lysates from INS cells (right). The hydrolysis of S223-AM was followed over time by HPLC and the peak integrals of S223-AM and the products S223 and OXO were plotted. The decay of S223-AM and the appearance of the products were fitted to single exponential kinetics shown as solid lines. (**B**) The fraction of S223 in the total product (S223 and OXO) after exposure of S223-AM to different media. The thin grey bars represent the expected fraction of S223 if OXO had been formed with the same rate constant as that observed in PBS. (**C**) Summary of the kinetic parameters determined for the hydrolysis of S223-AM in different media. Full kinetics in PBS, culture supernatant and lysates from INS cells that allowed the accurate determination of the rate constants of the decay of S223-AM (*k*_decay_) and the formation of S223 (*k*_S223_) and OXO (*k*_OXO_) was recorded as shown in (**A**). Limited kinetics that allowed an estimation of *k_decay_* were recorded for the remaining conditions. The half-life of S223-AM τ_1/2_ were calculated from *k_decay_*. PBS, phosphate buffer saline; Acetylesterase, 1 unit orange skin acetylesterase in PBS; RPMI, RPMI medium supplemented with foetal calf serum; supernatant, RPMI medium supplemented with serum taken from a culture dish with U2OS cells after 3 day of culture; U2OS, HUVEC, HEK293T, and INS, lysates of respective cells.

To characterise the stability and cleavage preferences, S223-AM was exposed to different media and its hydrolysis was monitored by HPLC. If dissolved in aqueous buffer (25% acetonitrile in PBS, pH 7.4), S223-AM decayed with a half-life of 4.5 h, whereby the undesired OXO was formed to about 97% (Figure 2). The stability of S223-AM in aqueous solutions is thus limited but in principle, sufficient for typical applications in tissue culture experiments. If acetylesterase was added at a concentration at which the reaction was completed in about 5 min, about 99% of the formed product was the desired S223 (Figure 2B,C). Thus, in the conditions under which the obtained product was almost exclusively formed via the enzymatic reaction and not by chemical hydrolysis, S223 was rapidly and efficiently released.

In RPMI tissue culture medium supplemented with heat inactivated (56 °C, 30 min) foetal calf serum, the half-life of S223-AM was reduced to about 15 min and 90% of the generated product was OXO. A further reduction to 8 min was observed in medium taken from a dish of cultured U2OS cells, whereby 95% of the generated product was OXO. This combination of short half-life and the high fraction of the undesired OXO as generated product was unexpected. The short half-lives suggest that the majority of the product was generated by an enzymatic reaction as the reaction velocity of chemical hydrolysis is expected to be similar to that observed in PBS. Heat inactivated calf serum seems, therefore, a source of considerable enzymatic activity that converts S223-AM to OXO. As this activity was increased in medium in which U2OS cells had been grown, it seems that the enzyme(s) is either actively secreted by U2OS cells or released by dying cells (Figure 2).

As a model to investigate the conversion process of S223-AM in cells, concentrated lysates of U2OS, HEK293T or INS cells or HUVEC were used (Figure 2). In these lysates S223-AM decayed with half-lives of less than 1 h. The product fraction of OXO varied between about 70% in HUVEC lysates and about 30% in INS cell lysates. Under these conditions, the majority of the product is thus formed by two enzymatic activities, one generating the desired S223 and one generating the undesired OXO. 

### 3.3. Characterisation of S223-AM in Living Cells

**Figure 3:**
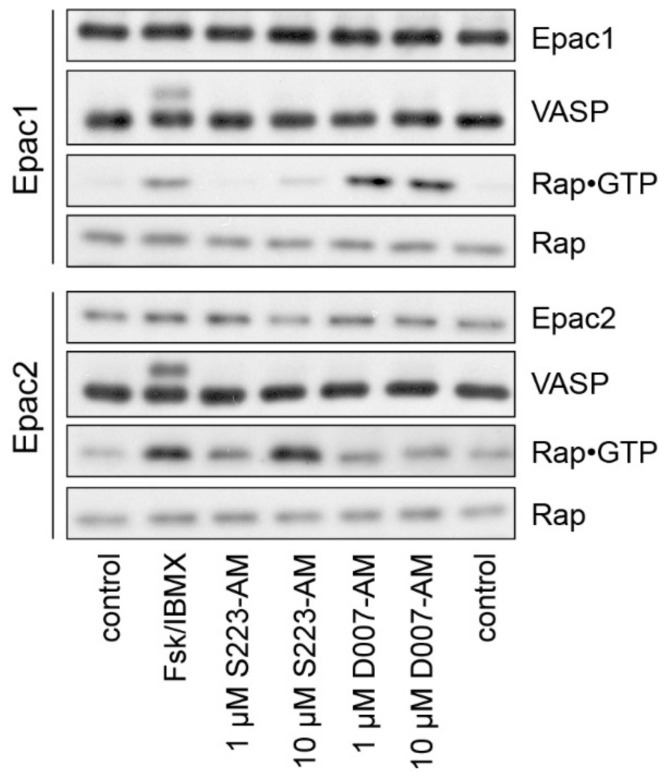
Rap and PKA activation in living cells. U2OS cells stably expressing Epac1 or Epac2 were stimulated as indicated. The activation of PKA was monitored by a phosphorylation-induced band shift of VASP. Rap•GTP was precipitated from cell lysates and compared to the total Rap levels. control, mock-stimulated; Fsk/IBMX, 15 μM forskolin and 200 μM IBMX to elevate intracellular cAMP levels.

U2OS cells do not express endogenous Epac1 and Epac2 but respond to increased cellular cAMP levels with PKA activation. Cell lines stably expressing either Epac1 or Epac2 were therefore established as a model system to assess the selectivity of cAMP analogues [30]. PKA activity was monitored by a phosphorylation-dependent band shift of vasodilator stimulated phosphoprotein (VASP), a substrate of PKA, and Epac1 or Epac2 activity by the specific precipitation of GTP-bound Rap. Cells were either stimulated with a combination of the adenylyl cyclase activator forskolin and the phosphodiesterase inhibitor IBMX to increase cellular cAMP levels, with D007-AM or with S223-AM (Figure 3). Forskolin/IBMX induced activation of PKA, Epac1 and Epac2. D007-AM and S223-AM selectively activated Epac1 and Epac2, respectively, presumably via intracellular conversion to the active species D007 and S223. 

### 3.4. S223-AM Selectively Activates Epac2 in β-cells

The potential use of S223-AM as a tool to investigate the function of Epac2 requires the characterisation of the selectivity of S223-AM in relevant cellular systems. The insulin-secreting β-cell line MIN6 was chosen since Epac2 has been implicated in insulin secretion, and these cells are well characterized in terms of cAMP signalling using fluorescence-based reporters for real time analysis at the single-cell level [11,21,22,35]. 

**Figure 4:**
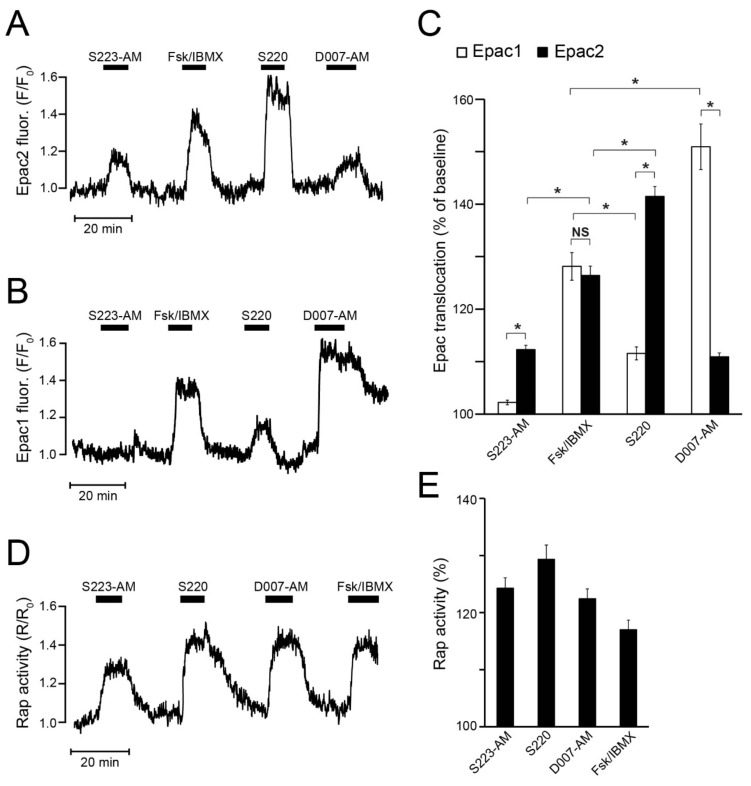
Translocation of Epac1 and Epac2 and activation of Rap at the plasma membrane in MIN6 β-cells. (**A**) TIRF recording of the change of plasma membrane fluorescence of GFP-Epac2 in a single MIN6 β-cell exposed to S223-AM (5 µM), S220 (100 µM), D007-AM (5 µM) and the combination of forskolin (fsk; 10 µM) and IBMX (100 µM). Representative for 75 cells from four experiments with three cell preparations. (**B**) Similar recording from a cell expressing YFP-Epac1. Representative for 54 cells from five experiments with three cell preparations. (**C**) Means ± s.e.m. for the Epac translocation responses to the different drugs expressed as time-averaged fluorescence intensity in per cent of the baseline. * *p* < 0.001 for indicated differences. Statistical comparisons were made with a Student’s *t*-test. (**D**) Changes of plasma membrane Rap activity recorded with TIRF microscopy from a single MIN6 β-cell as the GFP-RalGDS_RBD_/td2-CAAX fluorescence ratio. Representative for 25 cells from three experiments with three cell preparations. (**E**) Means ± s.e.m. for the effects of the drugs on Rap activity expressed as time-averaged fluorescence ratio normalized to the baseline. All the treatments had significant effects (*p* < 0.001, Student’s *t*-test).

Activation of Epac1 and Epac2 was monitored in MIN6 cells by imaging the cAMP-induced translocation of fluorescent-protein-tagged Epac versions from the cytoplasm to the plasma membrane using TIRF microscopy. In support of Epac2 selectivity, S223-AM induced prompt translocation of Epac2, but not of Epac1 (Figure 4A–C). S220 induced an even stronger Epac2 translocation than S223-AM, but also stimulated translocation of Epac1. D007-AM induced Epac2 translocation almost to the same extent as S223-AM but displayed a much more pronounced effect on Epac1 translocation (Figure 4A–C). Stimulation with forskolin/IBMX resulted in translocation of Epac1 and Epac2 to a similar extent. The effect of forskolin/IBMX was stronger than the effects of S223-AM and D007-AM on Epac2 translocation and that of S220 on Epac1 (Figure 4C). However, D007-AM and S220 were significantly more efficient to translocate Epac1 and Epac2, respectively, compared to forskolin/IBMX, consistent with these cAMP analogues being “superactivators” of Epac. Taken together, D007-AM and S220 showed a strong preference but not full selectivity, for Epac1 and Epac2, respectively, whereas S223-AM appeared to be Epac2-selective.

### 3.5. S223-AM Promotes Rap Activation at the β-Cell Plasma Membrane

As read-out of endogenous Epac activity, the formation of Rap•GTP was recorded in the MIN6 cells, although this assay does not allow discrimination between Epac1 and Epac2 activity. Since Rap is localised at the plasma membrane, Rap•GTP loading can be monitored by the recruitment of GFP-RalGDS^RBD^ that binds to the GTP- but not GDP-bound form of Rap. The application of S223-AM, S220, or D007-AM all induced immediate and fast Rap activation to a comparable extent, somewhat exceeding the effect of forskolin and IBMX (Figure 4D,E).

### 3.6. S223-AM Does Not Activate PKA in β-Cells

**Figure 5:**
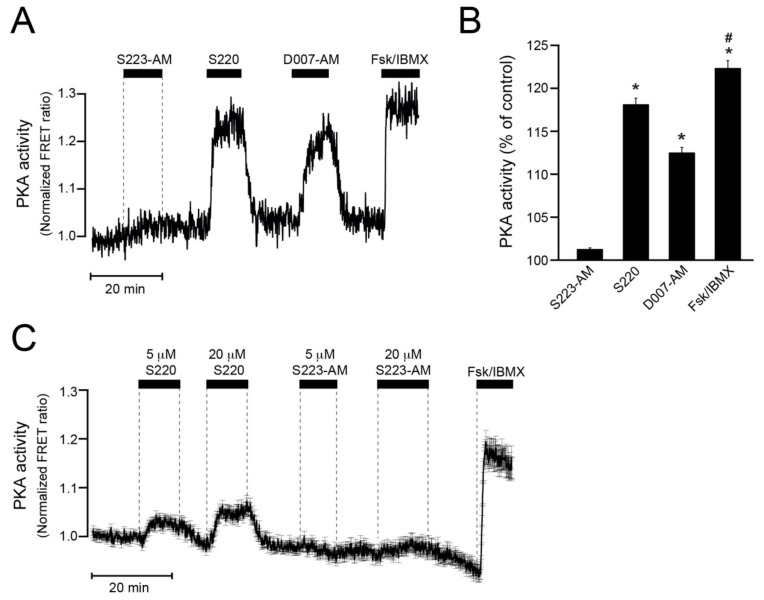
Recordings of PKA activity in MIN6 β-cells. (**A**) Representative single-cell time-lapse recording of PKA activity using TIRF microscopy and the PM-AKAR3 reporter. The cell was sequentially exposed to S223AM (5 µM), S220 (100 µM), 007AM (5 µM), and the combination of forskolin (10 µM) and IBMX (100 µM) added to the superfusion medium in the presence of a sub-stimulatory glucose concentration (3 mM). Increased PKA activity results in increased intramolecular FRET recorded as sensitized emission and expressed as normalized FRET ratio. (**B**) Means ± s.e.m. for the time-average FRET ratios during exposure to the different drugs normalized to control. N = 75 cells from three experiments with three different cell preparations. * *p* < 0.001 for difference from S223-AM; # *p* < 0.001 for difference from D007-AM and S220 (Student’s *t*-test). (**C**) Average ± s.e.m. of 78 single-cell FRET ratio traces from two recordings of PKA activity in MIN6 cells exposed to 5 and 20 µM of S220 and S223-AM as well as the combination of 10 µM forskolin and 100 µM IBMX.

The potential ability of the drugs to activate PKA was investigated by monitoring PKA activity in MIN6 cells using the PM-AKAR3 sensor, which shows increased intramolecular FRET upon phosphorylation by PKA. Whereas 5 µM S223-AM had little effect, 100 μM S220 and 5 μM D007-AM induced marked increases of PKA activity, although not as pronounced as forskolin/IBMX (Figure 5A,B). The difference between S220 and S223-AM was not due to the different extracellular concentrations employed. Accordingly, weak PKA activation was observed already at 5 µM S220, while there was no obvious effect of S223-AM even after increasing the concentration to 20 µM (Figure 5C).

### 3.7. cAMP-Dependent Rap Activity in β-cells is Mediated by Epac2

**Figure 6:**
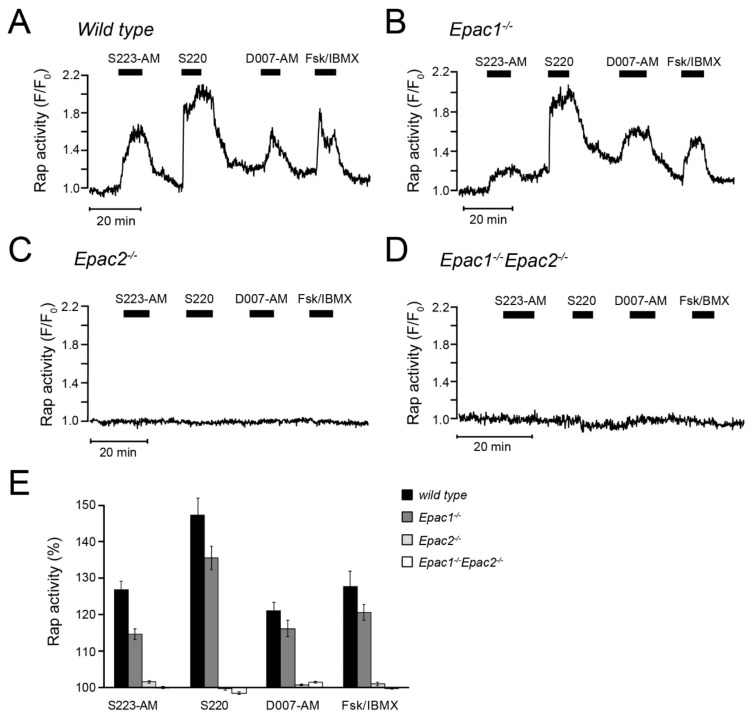
Changes of plasma membrane Rap activity in primary β-cells from wildtype and Epac-deficient mouse islets. (**A**) Single-cell TIRF microscopy recording from a wildtype islet transduced with GFP-RalGDS^RBD^. Representative for 37 cells from five experiments and four independent islet isolations. (**B**–**D**) Similar recordings from β-cells isolated from Epac1^-/-^ (**B**) Epac2^-/-^ (**C**) and Epac1/2-double knockout mice (**D**). Representative for 35 (**B**), 47 (**C**) and 75 (**D**) cells from four to five experiments and three independent islet preparations from each genotype. (**E**) Means ± s.e.m. for the effects of the Epac agonists on Rap activity expressed as time-averaged GFP-RalGDS^RBD^ fluorescence normalized to the baseline.

The cAMP analogues were further characterized by monitoring Rap activation with TIRF microscopy and the GFP-RalGDS^RBD^ assay in superficially located cells in intact pancreatic islets isolated from wild-type, Epac1^-/-^, Epac2^-/-^, or Epac1/2 double knock-out mice. S223-AM, S220, D007-AM and IBMX/forskolin all induced pronounced Rap activation in wildtype islet cells (Figure 6A). In Epac1^-/-^ cells, the effects of D007-AM and forskolin/IBMX were unaffected, whereas that of S220 and S223-AM was reduced by 30%–40% (*p* < 0.05) (Figure 6B,E). In contrast, no Rap activation was observed in cells from the Epac2^-/-^ or double knock-out mice (Figure 6C–E) irrespective of the stimulus. These observations strongly indicate that Rap activation in β-cells is mediated by Epac2 but not Epac1. 

## 4. Discussion

The development of cAMP analogues with selectivity profiles towards either of the two Epac proteins or PKA is important to improve the understanding of cAMP signalling in various biological systems. Apart from achieving specificity, it is a challenge to make poorly membrane-permeable nucleotides effective in living cells. For example, one of the most recently developed analogues, S223, shows excellent selectivity for Epac2 over Epac1 and PKA in vitro, but had little or no effect when tested in intact cells [30]. Here, we synthesised S223-AM as a prodrug and thereby transferred a well-established strategy to improve membrane permeability of phosphate-containing molecules to a thiophosphate. The ester linkage was exclusively formed with the sulphur and thus, as discussed in the Results Section, either S223 or the undesired OXO can be formed upon hydrolysis. In cell lysates, enzymatic activities that catalyse the formation of both reaction products were found. The relative proportion of formed S223 and OXO depended on the cell type.

Irrespective of this complication, we show that the conversion of S223 into a prodrug enables its use in living cells. S223-AM selectively activated Epac2 but not Epac1 or PKA in U2OS cells. This conclusion was corroborated by online recordings from single β-cells expressing fluorescent Epac constructs or reporters for Rap or PKA activity. S223-AM stimulated Epac2 translocation and Rap activity rapidly and without delay. S223-AM was also found to selectively activate Epac2 but not Epac1 or PKA in β-cells. The capability of S223-AM to activate Epac2 remained lower than that of S220. This is in agreement with the biophysical characteristics of S220 as a stronger Epac2 agonist than S223 [30]. However, in contrast to S220, S223-AM did not activate PKA in β-cells. S223-AM is thus superior to S220 for the use in cells, if the activation of Epac2 but not Epac1 or PKA is desired. The rather strong PKA activation in β-cells caused by S220 was unexpected as little or no PKA activation was previously reported employing U2OS cells [30]. However, S220-induced activation of PKA is supported by biophysical characterisation of the analogue, showing that depending on the PKA isoform, the affinity of S220 for PKA is similar or only slightly reduced compared to that of cAMP [30]. 

In view of previous studies, it was surprising that D007-AM induced activation of PKA in β-cells. In agreement with its reduced affinity for PKA, several studies in cellular systems report no activation of PKA upon application of either D007 or D007-AM [28,30,32]. There are several potential explanations for this discrepancy. For instance, detection of PKA activity in real time by TIRF microscopy is probably more sensitive than assays based on antibodies and Western Blotting. cAMP analogues may also antagonise cAMP degrading phosphodiesterases (PDEs) [36], the group of enzymes that catalyse the degradation of cAMP [1]. The observed PKA activation may thus in part be caused by increased cellular cAMP levels due to inhibition of PDEs. The magnitude of this effect could vary between cell types depending on the expression pattern of PDEs and the effect of the analogue on the particular PDE enzyme. Along the same lines, the cAMP analogue may have dissimilar effects on different PKA regulatory subunits and differences between cells may reflect diverse expression levels of particular PKA isoforms. The extent of side-effects may obviously also be a matter of concentration. Since the hydrolysis of the prodrug will lead to intracellular accumulation of the cAMP analogues, it is difficult to precisely control the intracellular concentration. D007-AM enters cells very efficiently and induces Epac activation in HUVEC cells starting at a concentration of 10 nM, which is about three orders of magnitude lower than the required concentration of D007 [32]. In the present study, D007-AM was applied at a concentration of 5 µM, and it is possible that D007 may reach an intracellular concentration that is sufficient for PKA activation. Importantly, S223-AM were without effect on PKA even at a concentration of 20 µM, indicating a higher specificity of this analogue.

We found that cAMP-stimulated Rap activation at the plasma membrane was completely abolished in pancreatic islet cells from Epac2 knockout mice. This finding indicates that plasma membrane Rap activity is entirely dependent on Epac2, which is in agreement with a low Epac1 expression in β*-*cells [13,14]. Wildtype and Epac1-deficient cells readily responded to endogenous cAMP elevation as well as to the different cAMP analogues. Unexpectedly, the responses to S223-AM and S220 were somewhat reduced in Epac1-deficient cells. The explanation for this effect is unclear, but it is not likely to reflect an action of these compounds via Epac1, since the responses to 007-AM and cAMP elevation with confirmed effects via Epac1 were unaffected. Moreover, if Epac1 would have contributed to Rap activation, some Rap activity would be expected to remain in the Epac2-deficient cells.

Several studies on insulin secretion have used D007-AM or D007 as a cAMP analogue for activation of both Epac1 and Epac2, but not PKA. We believe that the data presented here may help to refine the interpretation of some of these earlier experiments. It is clear from the biophysical characterisation of D007, that the analogue is more potent than cAMP in activating Epac1 but less potent than cAMP in activating Epac2 [26,30]. In agreement with these findings, D007 was found to be a poor activator of Epac2 in the U2OS test system [30]. These observations were confirmed here using β-cells. D007-AM induced a stronger Epac1 response than elevation of cAMP levels by forskolin/IBMX, whereas the Epac2 response to D007-AM is weaker than that to forskolin/IBMX. The D007-induced effects previously reported in β-cells may thus well be Epac2 mediated. However, as discussed above, D007-AM is also able to activate PKA in β-cells. It is thus crucial to exclude a putative contribution of PKA to effects that previously have been attributed to Epac2 in β-cells based on experiments with D007. Clarifying experiments would now be possible by the use of S223-AM. With its promising properties regarding both Epac2 selectivity and membrane permeability, S223-AM should become an important tool for investigating cAMP effector involvement not only in β-cells and insulin secretion, but also in many other processes in various types of cells.

## Figures and Tables

**Table 1:** Assignment of the ^1^H-, ^13^C-, and ^31^P-NMR spectra of S223-AM.

Atom	^31^P	^13^C	^1^H		
	/ppm	/ppm	/ppm	Multi-plicity	*J*/Hz
P	19.92				
2		155.2	8.202	s	
4		153.5			
5		122.5?			
6		157.6			
8		150.1			
6N’			7.330		
6N’’			6.788		
1’		92.75	6.001	s	
2’		83.31	4.73	d	5.3
3’		81.06	5.752	m	5.1/5.3
4’		73.30	4.335	td	4.9/10.3
5’		73.88	4.828	dq	4.9/23.3
5”			4.412	t	10.1
L1		39.50	4.679	d	3.2
L2		140.1			
L3		132.3	7.561	d	8.0
L4		131.52	7.422	t	7.3
L5		130.66	7.377	t(d)	7.3 (2.0)
Z1		61.37	3.521	s	
A1		23.9	2.177	s	
A2		172.8			
A3′		65.0	5.653	dd	11.2/21.2
A3′’			5.571	dd	11.2/18.8

d, doublet; dd, double doublet; dq, double quadruplet; m, multiplet; s, singlet; t, triplet; td, triple doublet.
